# Endoscopic removal of proximally migrated stents using a double‐balloon enteroscope in patients with bowel reconstruction (with video)

**DOI:** 10.1002/deo2.32

**Published:** 2021-09-07

**Authors:** Takashi Oda, Kazuyuki Matsumoto, Eijiro Ueta, Hitomi Himei, Taiji Ogawa, Hiroyuki Terasawa, Yuki Fujii, Tatsuhiro Yamazaki, Shigeru Horiguchi, Koichiro Tsutsumi, Hironari Kato, Hiroyuki Okada

**Affiliations:** ^1^ Department of Gastroenterology Okayama University Hospital Okayama Japan

**Keywords:** double‐balloon enteroscope (DBE), stent migration, stent removal

## Abstract

Endoscopic migrated stent removal using a balloon‐assisted enteroscope is technically difficult in patients with bowel reconstruction. We report the treatment outcomes and endoscopic removal methods for migrated stents using a double‐balloon enteroscope (DBE). We retrospectively studied 12 patients with stent migration into the main pancreatic duct (MPD) or bile duct who underwent bowel reconstruction between January 2012 and June 2020. The successful removal rates in the MPD (*n* = 3) and the bile duct (*n* = 9) were 66.7% (2/3) and 88.9% (8/9), respectively. The removal techniques included the indirect method (*n* = 3), the direct method (*n* = 4), and a combination of indirect and direct methods (*n* = 3). The removal devices included an extraction balloon catheter (*n* = 7), basket catheter (*n* = 5), biopsy forceps (*n* = 3), and snare (*n* = 2). Stent removal using a DBE was feasible and useful as the first treatment for patients with bowel reconstruction. The choice of the direct and/or indirect method according to the situation of the migrated stent is important.

## INTRODUCTION

Internal stenting across a pancreaticojejunostomy (PJ) or hepaticojejunostomy (HJ) during surgery followed by pancreaticoduodenectomy (PD) or hepatectomy may be useful for preventing pancreatic fistula or bile leakage. Endoscopic stenting for anastomotic stricture using a balloon‐assisted endoscopy is also performed widely for patients with surgically altered gastrointestinal anatomy. However, stent migration occurs and sometimes causes conditions such as cholangitis, bile duct stones, pancreatitis, and pancreatic stones.^1^, ^2^, ^3^ Park et al.^3^ reported that the incidence of internal PJ stent migration into the bile ducts after PD was 16.8% (135/802). Among them, 29.6% (40/135) of patients showed stent‐induced complications. For cases with bowel reconstruction, the endoscopic removal of migrated stents is more technically difficult and has rarely been reported. We herein report the outcomes and endoscopic removal methods for migrated stent in patients with bowel reconstruction using a double‐balloon enteroscope (DBE).

## CASE REPORT

### Patients

We studied 12 patients with stent migration into the main pancreatic duct (MPD) or bile duct who underwent bowel reconstruction between January 2012 and June 2020. All patients presented with symptoms, such as cholangitis and pancreatitis, due to stent migration. Before the DBE procedure, stent migration was confirmed by X‐ray and computed tomography (CT). All participants provided written informed consent, and this study was approved by the institutional review board of our hospital.

### Endoscopic stent removal methods

**FIGURE 1:**
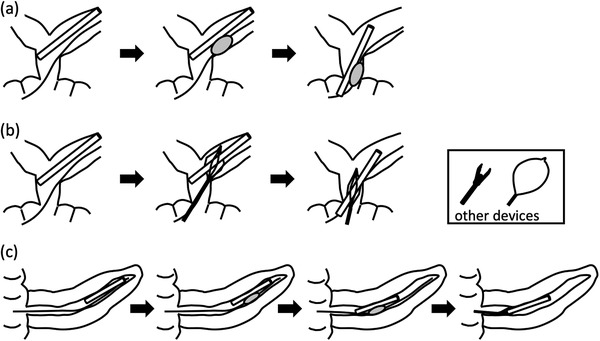
(a) Schematic illustration of the removal of a migrated bile duct stent using the indirect method. (b) Schematic illustration of the removal of a migrated bile duct stent using the direct method. (c) Schematic illustration of the removal of a migrated pancreatic duct stent using a combination of indirect and direct methods

We used a short‐type DBE (EC‐450BI5, EI‐530B; working length 152 cm, working channel 2.8 mm, or EI‐580BT; working length 155 cm, working channel 3.2 mm; Fujifilm) and an overtube (TS‐13101; working length 105 cm; Fujifilm). The stent was removed by the indirect and/or direct method, which is described in detail below and illustrated in Figure 1.

#### Indirect method

Indirect traction is applied with an extraction balloon (TRI‐EX Triple Lumen Extraction Balloon; Cook or Multi‐3; Olympus). The extraction balloon is positioned alongside or in the stent and is inflated until the stent and balloon adhere to each other, and then the balloon is carefully pulled. After all or part of migrated stent comes out on the bowel side with balloon traction, the stent is picked up with a forceps. If the proximal stricture of the MPD or bile duct is severe, balloon dilation (REN Biliary Dilation Catheter; Kaneka Co. or ZARA EPBD balloon; Century Medical) is performed before stent removal (Figure 1a). When the migrated stent was located at the far side of the anastomosis site, the indirect method was first chosen for stent removal.

#### Direct method

Direct traction is applied with various devices, including forceps (Radial Jaw 4 Biopsy Forceps; Boston Scientific), basket (Memory eight‐wire type outer diameter 8Fr; Cook), or snare (Captivator; Boston Scientific). The retrieval technique using forceps is carried out by directly grasping the end or side of the stent or a flap of the stent. Retrieval using a basket or snare is performed by directly grasping the distal end of the stent. A basket has multiple wires and can therefore capture the stent more easily than a snare (Figure 1b).

#### Combination of indirect and direct methods

First, the indirect method is used to move the stent from the distal side to the proximal side with an extraction balloon. Subsequently, the direct method (e.g., a forceps, basket, or snare) is used to grasp and remove the stent (Figure 1c).

## RESULTS

Table 1 shows the characteristics and treatment outcomes of 12 patients. The median age was 71.5 (63–79) years old and the bowel reconstruction methods were the modified Child method in seven and Roux‐en‐Y in five patients. The reasons for the initial stent placement were internal stenting for the HJ or PJ site during surgery in six cases (HJ: 4, PJ: 2), stricture at the anastomosis site in five cases (HJ: 4, MPD: 1), and stricture at the intrahepatic bile duct in one case. The median duration from stent placement to DBE procedure was 165 days (range, 28–1066 days).

**TABLE 1:** Characteristics of patients and proximally migrated pancreatic and bile duct stents, including the endoscopic retrieval techniques used in our study

Migrated stents	Case	Age (years) /sex	Disease	Surgery	Bowel reconstruction method	Type of migrated stents	Size of stent (Fr)	Length of stent (cm)	Stricture at anastomosis site	Location of the migrated stent	Diameter of MPD or bile duct (mm)	Scope	Removal techniques	Attempted removal techniques (use turn order)	Technical outcome	Procedure time (mm)
Pancreatic duct stents	1	71/M	Gastric cancer	Total gastrectomy	Roux‐en‐Y	Straight with flap	5	12	N.A.^†^	MPD	3	EI‐580BT	Indirect + Direct^‡^	Balloon extraction / Basket	Success	43
	2	75/F	PDAC	SSPPD	Modified Child	Lost stent	6	5	Yes	MPD	5	EI‐580BT	Indirect + Direct	Balloon extraction / Basket / Direct forceps	Success	30
	3	63/M	BDCa	SSPPD	Modified Child	Lost stent	6	4.5	Yes^§^	MPD	6	EC‐450BI5	None	None	Failure	32
Bile duct stents	4	75/M	IPMC	TP	Roux‐en‐Y (hepaticojejunostomy)	Straight with flap	7	5	Yes	B2	5	EI‐580BT	Indirect^¶^	Balloon extraction	Success	12
	5	70/F	IPMC	PD	Modified Child	Lost stent	6	6	Yes	B2	11	EI‐580BT	Direct	Basket	Success	3
	6	71/F	IPMC	PD	Modified Child	Straight with flap	7	7	No	B2	10	EI‐580BT	Indirect	Balloon extraction	Success	6
	7	77/M	Duodenal cancer	SSPPD	Modified Child	Straight with flap	7	5	No	B2	7	EI‐580BT	Direct	Direct forceps	Success	9
	8	70/F	PDAC	PD	Modified Child	Straight with flap	7	7	No	B2	4	EI‐580BT	Indirect	Balloon extraction	Success	20
	9	79/M	CCC	Hepatectomy	Roux‐en‐Y	Lost stent	6	3 and 5	No	B8, B3	4, 5	EI‐530B	Indirect + Direct	Balloon extraction / Basket / Direct forceps	Success	60^††^
	10	72/M	CCC	Hepatectomy	Roux‐en‐Y	Lost stent	6	4	No	B8	2	EI‐580BT	Direct	Snare / Basket	Success	38
	11	66/M	GBCa	Hepatectomy	Roux‐en‐Y	Lost stent	5	5	No	B6	4	EI‐580BT	None	Balloon extraction	Failure	16
	12	76/M	BDCa	SSPPD	Modified Child	Straight with flap	7	5	No	Right hepatic duct	5	EI‐530B	Direct	Snare	Success	9

Abbreviations: BDCa, bile duct cancer; CCC, cholangiocellular carcinoma; GBCa, gallbladder cancer; IPMC, intraductal papillary mucinous carcinoma; MPD, main pancreatic duct; PD, pancreaticoduodenectomy; PDAC, pancreatic ductal adenocarcinoma; SSPPD, subtotal stomach‐preserving pancreaticoduodenectomy; TP, total pancreatectomy.

^†^ Naive papilla.

^‡^ Indirect + direct method means that the stent was moved to the proximal side using a balloon, and the stent was removed switching to the direct method using a forceps, basket, or snare.

^§^ The site of pancreaticojejunal anastomosis could not be detected by DBE‐ERCP.

^¶^ Indirect method means that the stent was moved from the anastomotic site to the intestinal tract using a balloon alone.

^††^ Time required to remove two stents.

**FIGURE 2:**
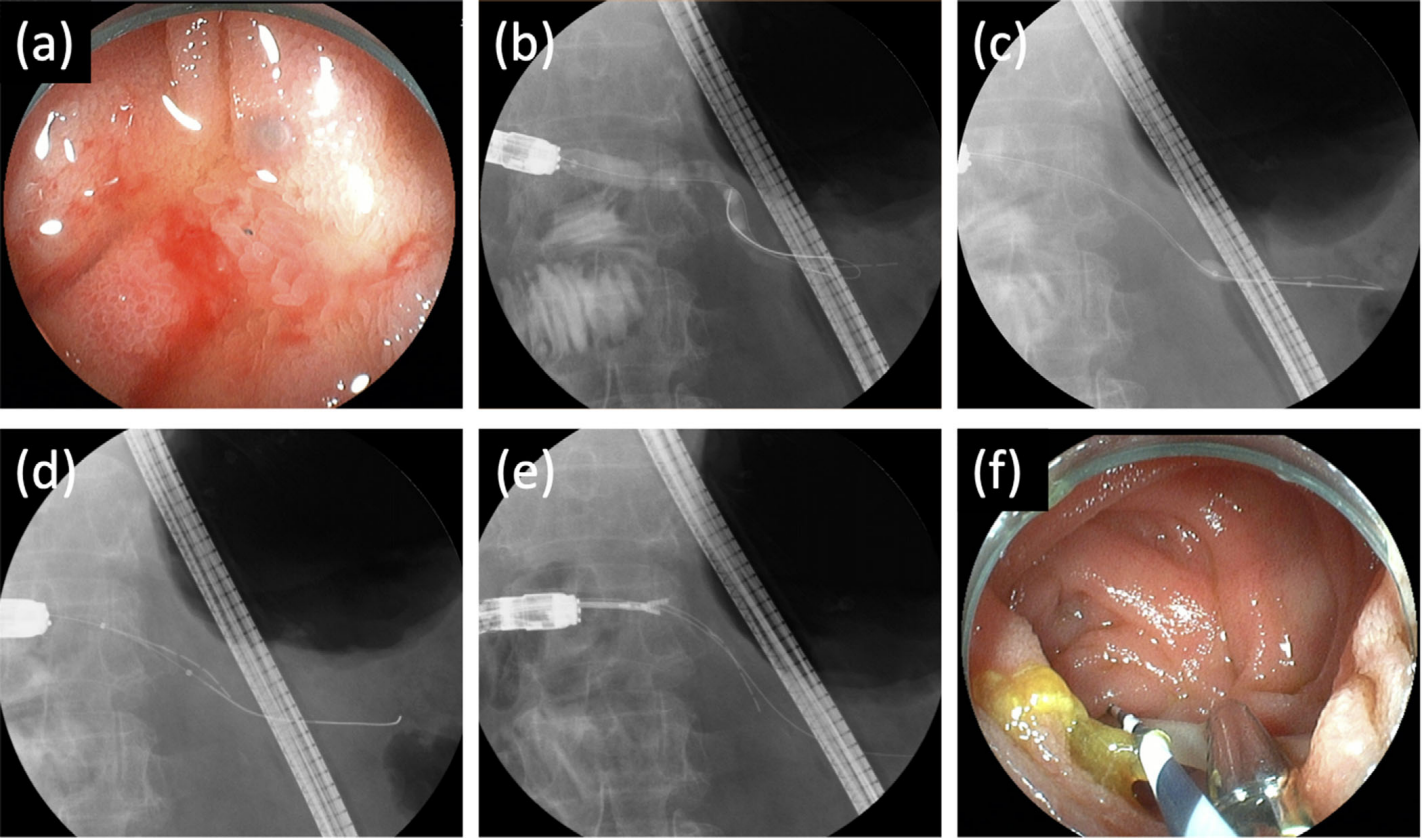
(a) The anastomotic site was narrowed, like a pinhole. (b) The balloon was inflated to dilate the stenosis. (c) The balloon was positioned alongside the internal stent and then inflated. (d) The stent was pulled down distally by indirect traction using a balloon catheter. (e) The side of the stent was grasped directly using forceps. (f) The stent was successfully removed

The indications for pancreatic duct stent removal were two for pancreatitis and one for pancreatic stones. The successful removal rate in the MPD was 66.7% (2/3), and the median procedure time was 32 min (range, 30–43 min). In two patients with successful stent removal, balloon extraction was initially used and the position of the migrated stent was changed to the proximal side. Subsequently, the stent could be removed with a basket and direct forceps. We show a figure and video of cases of successful pancreatic stent removal (no. 1: Video S1; no. 2: Figure 2).

**FIGURE 3:**
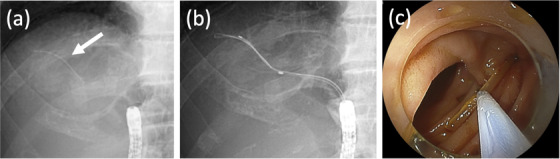
(a) The internal stent (white arrow) that migrated into the bile duct was identified on an X‐ray film. (b) The stent was caught by direct traction using a basket. (c) The stent was collected using a snare, after a part of the stent was pulled down into the small intestine

The indications for bile duct stent removal were eight for cholangitis and one for intrahepatic stones. The successful removal rate in the bile duct was 88.9% (8/9), and the median procedure time was 12 min (range, 3–60 min). In three of nine (33.3%) patients, the stent could be removed by indirect balloon extraction. Direct traction was used in five (55.6%) patients, including a forceps in two, a basket in two, and a snare in one. We show a figure and video of the case of successful bile duct stent removal (no. 10; Figure 3 and Video S2). All of the procedures were performed without any procedure‐related complications.

The stents reimplantation was performed in four cases with anastomosis stricture (HJ: 3, PJ: 1), and the pig‐tail‐type stent or a long stent (7–9 cm) was selected to prevent migration.

## DISCUSSION

In our case series, the rates of overall successful endoscopic retrieval using a DBE in patients in whom the internal stent migrated into the MPD and the bile duct after bowel reconstruction were 66.7% and 88.9%, respectively. Stent removal using a DBE was a feasible procedure that should be considered as the first treatment in patients with stent migration.

Three reports on endoscopic removal of migrated lost stents after PD using a balloon enteroscope have been described (Table S1).^4^, ^5^, ^6^ These reports included six cases with migration into the bile duct^4^, ^6^ and one case with migration into the MPD.^5^ Six of the cases were treated using a DBE^5^, ^6^ and one was treated using a single‐balloon enteroscope.^4^ In all reports, the stent was successfully removed using a basket without complications. Our study included all patients in whom removal of a migrated stent was attempted, and endoscopic stent removal was effective in 10 of the 12 cases. In one unsuccessful case, the site of PJ anastomosis could not be detected. In the other case, the stent migrated into a severely bent posterior bile duct branch. Fortunately, those cases were managed by conservative therapy with antibiotics. If there were no improvements, we had performed EUS‐guided pancreaticogastrostomy and percutaneous trans‐hepatic drainage for each case, and stent removal through the fistula would be attempted.^9^ Recently, the efficacy of fine‐gauge biliary balloon dilation catheters for stent removal has also been reported.^10^

Several reports have described the endoscopic removal of migrated pancreatic or bile duct stents in cases with normal anatomy.^7^, ^8^ Chaurasia et al.^7^ described a case involving the endoscopic removal of a migrated bile duct stent, and reported that the rate of overall successful endoscopic retrieval was 90% and that the stent could be removed by indirect balloon extraction in four of 41 (10%) cases and direct traction in 25 (61%) cases; the instruments were a forceps in three cases and a basket in 22. In DBE, the working channel is smaller and the scope length greater than with conventional endoscopic retrograde cholangiopancreatography (ERCP). Furthermore, the angulation of the scope can be strong in cases with the adhesion of the digestive tract after bowel reconstruction. These points limit the availability of devices for stent removal and reduce the operability of the scope. Therefore, retrieving migrated stents in bowel reconstruction using a DBE is technically difficult.

In conclusion, we described the treatment outcomes and endoscopic removal methods for migrated stents. Using basic methods (direct, indirect, and combination), migrated stents could be safely removed, even in patients with bowel reconstruction. Stent removal using a DBE was a feasible procedure that should be considered as the first treatment in patients with stent migration.

## CONFLICT OF INTEREST

The authors declare no conflict of interest.

## FUNDING INFORMATION

None.

## Supporting information


**Supplementary Table 1**. Summary of published data on endoscopic removal of migrated lost stents after PD using a balloon enteroscope

**Video S1**. Endoscopic removal of a migrated pancreatic stent by a combination of indirect and direct methods using an extraction balloon and a basket.

**Video S2**. Endoscopic removal of a migrated bile duct stent by the direct method using a basket. The internal stent that had migrated into the bile duct was identified on an X‐ray film. The bile duct was cannulated with a catheter, and a 0.025‐inch guidewire was advanced into the bile duct. We attempted to remove the stent with a snare; however, the axis did not match and we could not catch it. Thus, we used a basket (eight‐wire type). The basket was gradually opened, and we could catch the stent. The stent was collected using a snare, after part of the stent was pulled down into the small intestine.
